# Emergency central venous catheterization revisited

**DOI:** 10.4103/0974-2700.55348

**Published:** 2009

**Authors:** Sandeep Sahu, Indu Lata, Shikha Sachan, RK Singh

**Affiliations:** Department of Anaesthesiology, G.S.V.M. Medical College, Kanpur, U.P, India; 1 Department of Obstetrics and Gynaecology, V.M.M.C and Safdarjung Hospital, N. Delhi, India; 2 Department of Surgery, G.S.V.M. Medical College, Kanpur, U.P., India

Sir,

Internal jugular vein (IJV) catheterization is a fairly routine part of management of seriously ill patients and for the measurement of central venous pressure. In emergency department (ED) a resident doctor attempted to perform a right internal jugular vein canulation while managing a case of Carcinoma ovary. After cathertization the patient complained of respiratory distress and increasing chest discomfort. On examination, the patient was found to have decreased air entry in the right side of the chest and a dull note on percussion in the same side. frothy edematous fluid was aspirated through the central line port pink, came out. Suspecting misplacement of the catheter a chest X-ray was sought which showed a massive diffuse opacity in the right lung and incorrect position of the central catheter tip. The Right sided hydrothorax was managed by inserting a intercostals drain (ICD). This scenerio prompted us visit the topic of emergency central venous catheterization.

More than 5 million Central venous canulation (CVC) are placed each year in the United States, with an associated complication rate of >15%. Mechanical complications such as arterial puncture and pneumothorax are seen in up to 21%, and up to 35% of insertion attempts are not successful.[1,2] The risk of complications depends on several factors, including (but not limited to) operator experience, urgency of placement, as well as patient factors such as obesity, prior difficult cannulation and coagulopathy.[3,4] Moreover, accidental hydrothorax is more frequently associated with subclavian vein canulation and is a relatively uncommon in internal jugular vein canulation. Today the use of ultrasound has been associated with a reduction in complication rate and an improved first-pass success when placing catheters in the internal jugular (IJ) vein. From this case\ discussion, it would be prudent to remember that:-

1. Only a medical practitioner having enough experience with central venous cannulation should perform this procedure.
2. Aspiration of blood from the catheter port must always be done to confirm catheter placement in the right vessel.
3. A post-procedural chest X-ray must always be ordered.
4. Regular central venous pressure monitoring must be done.
5. Patients' complaint of rapid onset chest discomfort and distress should always be attended.

## Central venous access protocol

Line Sepsis is extremely serious and can be life threatening. All reasonable precautions must be followed to minimize sepsis rate. The following protocol is an inexpensive, effective system for placing new line and changing lines.[5]

1. All equipment must be at hand, transducers calibrated and the monitors functional prior to starting. It is the residents' responsibility to be sure this is done prior to beginning. Do not open kit until you are ready to perform procedure and you are certain you have the correct catheter.
2. Position the patient including placing EKG leads, supplemental oxygen and a pulse oximeter.
3. Lower the side rail.
4. Prepare the primary site and alternative site, usually the IJV and subclavian Vein using a 4 × 4 packs and Betadine.
5. Wash hands. Put on a cap, mask, gown, and gloves.
6. Open kit. Do a final prep with the prep sticks contained in the introducer or central access kit.
7. Apply the small fenestrated sheet directly over the site.
8. Apply the large fenestrated sheet contained in the universal access kit or a fenestrated utility sheet directly over the site. The field must be larger in all directions than the length of the wire. There should be no intravenous tubing hanging near the procedure site.
9. Infiltrate Lidocaine.
10. Place the patient in Trendelenburg position. In general, maximum Trendelenburg is used, but in some situations such as congestive heart failure, none is used.
11. Gain access to the vein and using the Seldinger technique, dilate the track and place the catheter or introducer. Usual insertion distance is 15 cm. Use a sterile extension tubing to attach the side arm of the introducer or the CVC to the intravenous solution after anchoring the catheter in place. A needleless anchoring device is effective, available and may decrease needle injuries.
12. Keep skin site sterile until final dressing is applied.
13. An X-ray is required to confirm placement, and absence of pneumothorax.

So, by taking care of all the above reasonable precautions while putting CVC lines in ED one can minimize catastrophes and complications with increase quality of care and better patient outcome.
